# The equine patellar ligaments and the infrapatellar fat pad – a microanatomical study

**DOI:** 10.1186/s12917-023-03579-3

**Published:** 2023-01-23

**Authors:** Cathrine Taule Fjordbakk, Patrick Marques-Smith

**Affiliations:** Faculty of Veterinary Medicine, Department of Companion Animal Clinical Sciences, Norwegian University of Life Sciences, Equine Teaching Hospital, Oluf Thesens Vei 24, 1432 Ås, Norway

**Keywords:** Patellar ligaments, Infrapatellar fat pad, Histology, Equine

## Abstract

**Background:**

Interpretation of patellar ligament (PL) ultrasonography may be difficult, as hypoechoic or heterogenous echogenicity are common findings. Verifying suspected disease of equine PLs by histopathology is also problematic as descriptions of normal PL vascularity and histology are scarce. The current study describes the PL and infrapatellar fat pad (IFP) vascular pattern from computed tomography scans of barium perfused normal equine specimens (*n* = 8; age 10 days to 18 years), as well as routine histology to serve as a reference for future investigations into PL pathology and IFP disease.

**Results:**

The PLs received a bipolar blood supply. Vascular architecture consisted of numerous distinct longitudinal vessels with several horizontal connections, which branched into extensive latticeworks of smaller vessels throughout the ligaments. Several vascular connections between the PLs and the IFP were identified. One distinct longitudinal vessel was seen entering each of the IFP lobes at the distocranial aspect, branching extensively into lobar vascular networks which anastomosed by several horizontal branches at the mid portion of the IFP where the two lobes merge. Histologically, there were large variations in PL interfascicular endotenon thickness, vascularity and fatty infiltration; these parameters increased with age for the intermediate and medial PL. Areas of metaplastic tenocytes / chondroid metaplasia were identified in all investigated adult medial PLs; in 2/7 in the intermediate PL and in 4/7 in the lateral PL. The adult IFP consisted of white unilocular adipose tissue, organized in lobules separated by thin connective tissue septa increasing in thickness towards the periphery and the distocentral aspect.

**Conclusions:**

The equine PLs and IFP are highly vascularized structures with ample vascular connections suggestive of crosstalk. This, together with the large variation in PL endotenon thickness, vascularity and fatty infiltration, should be taken into consideration when assessing potential PL histopathology as these changes increase with age and are found in horses without clinical signs of stifle disease. Metaplastic tenocytes / chondroid metaplasia should be considered a normal finding throughout the medial PL and is not age dependent. The role of the equine IFP in stifle disease has yet to be elucidated.

**Supplementary Information:**

The online version contains supplementary material available at 10.1186/s12917-023-03579-3.

## Background

Stifle injuries are increasingly recognized as a cause of hind limb lameness in equine practice. In the diagnostic work-up of a stifle lameness, ultrasonographic examination of the soft tissues including the patellar ligaments (PL) has become routine and is well described [1–5]. Whereas lateral PL lesions often are related to an external trauma [3], lesions of the intermediate PL may resemble the appearance of flexor tendon core lesions [1, 2] and a similar pathophysiology with progressive degeneration due to repetitive strain has been proposed [2]. However, interpretation of ultrasonographic findings of the PLs may be problematic, as ultrasonographic appearance does not always correspond to clinical signs. Ligament thickening, hypoechoic areas or areas of heterogenic echogenicity, all of which could be interpreted as pathological lesions, have been reported in the medial and the intermediate PLs of sound warmblood riding horses in full work [6]. Moreover, in a study of horses with ultrasonographic lesions of the intermediate PL, ultrasonographic appearance seemingly worsened during a period of rest even though the lameness improved as horses were treated for their concurrent intra-articular stifle pathology [5].

This apparent poor correlation between ultrasonographic and clinical findings is also a trait of the most common overuse condition of the human patellar tendon [7]. Patellar tendinopathy, or ‘jumper’s knee’, is characterized by load-dependent patellar tendon pain mainly affecting athletes of jumping sports [8]. Ultrasonographic findings such as thickening of the tendon and heterogenic tendon echogenicity are not always present and may also be found in non-clinical athletes [7]. However, neovascularization of the patellar tendon identified by power doppler ultrasonography has been identified as a more specific diagnostic indicator of clinical patellar tendinopathy [7], most likely as nociceptive nerve fibers accompany the blood vessel ingrowth [9]. Power doppler ultrasonography has also been used for detecting neovascularization in superficial digital flexor tendinopathy in horses [10] and could potentially be useful for diagnosing PL lesions. However, whereas the normal vascular architecture of the equine superficial digital flexor tendon has been described [11], such information is lacking for the equine PLs. Therefore, prior to considering neovascularization as an indicator of equine PL disease, description of the normal vascular architecture must be available.

There is also a paucity in the equine scientific literature regarding the infrapatellar fat pad (IFP), and its potential contribution to stifle disease. In human medical literature, IFP hypertrophy has been associated with patellar tendinopathy [12] and the IFP is increasingly recognized as a major contributor in pathological joint states such as anterior knee pain and osteoarthrosis [13]. Furthermore, the IPF is hypothesized to be an active component of the joint organ with multifunctional roles in the maintenance of joint homeostasis, as it contains immune cells, inflammatory cells and substance P nerve cells, all of which may play a role in disease development and could contribute to OA [13, 14].

The objectives of the current study were therefore to provide a detailed description of the vascular and histological architecture of normal equine PLs and the IFP to serve as a reference for future investigations into PL pathology and IFP disease.

## Results

### Ultrasonography

Ultrasonographic examination of the PLs of case 2 – 5 and case 8 were unremarkable; the ligament shape, size, echogenicity and fiber pattern were within what has been reported elsewhere as normal and were bilaterally symmetric [1–3, 6]. In case 6, the lateral PL had a bilateral symmetric heterogenic appearance at the tibial insertion with some anechoic areas observed in the transverse plane. Correspondingly, in the longitudinal plane, the ligamentous insertion appeared to have a fanlike pattern with hypoechoic areas interspersed between the ligament bundles. Case 7 had a pronounced mottled appearance of the intermediate PL at its tibial insertion which was bilaterally symmetric. Also, the origins of the lateral PLs were ill-defined in this horse. None of the included horses had abnormal findings of any other structure (menisci; collateral ligaments; femoral trochlea and trochlear groove; or synovial pouches). In all horses, the IFP was identified as an echogenic and heterogenic structure located between the PLs and the femoropatellar joint.

### CT scanning and vessel morphology

**Fig. 1:**
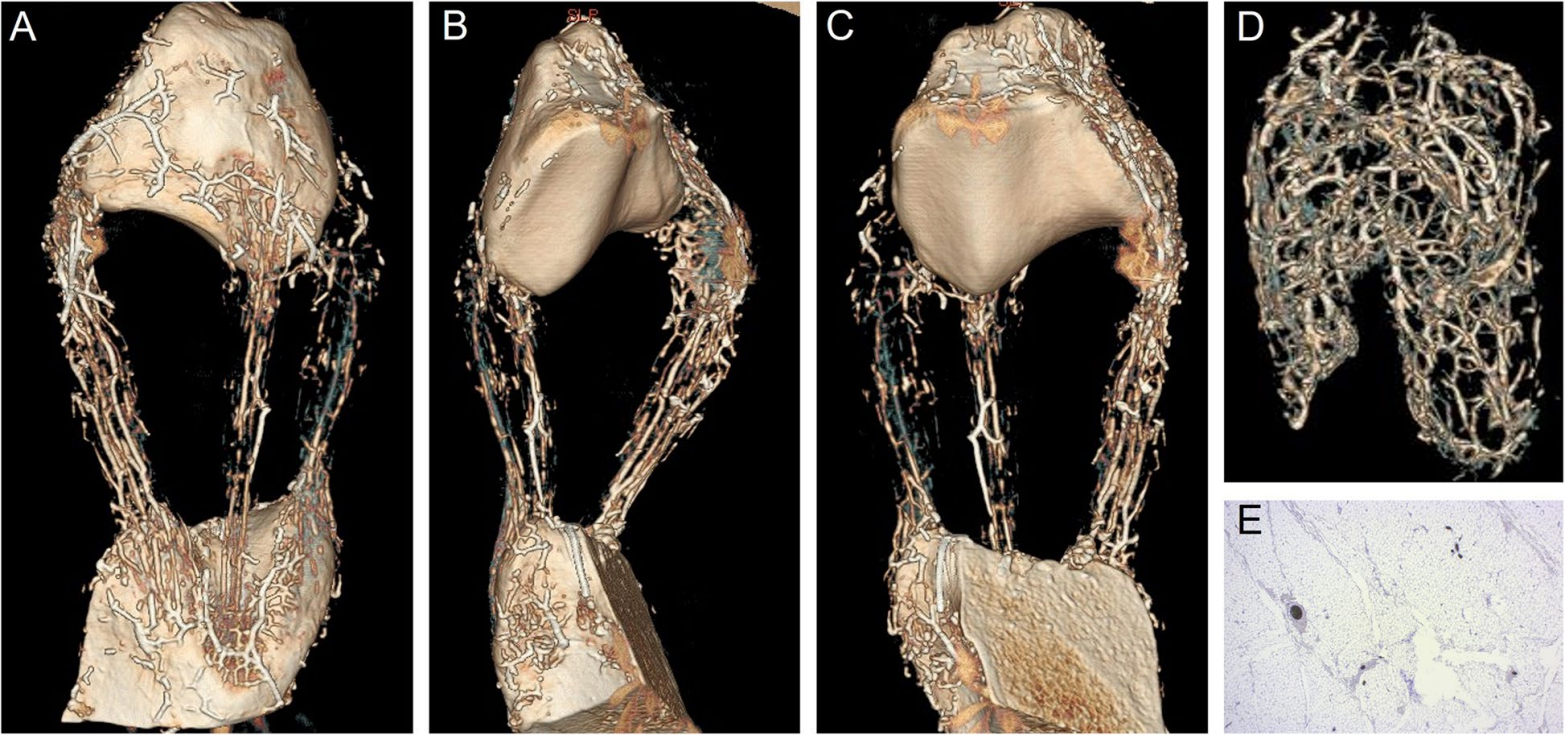
Patellar ligament and infrapatellar fat pad vascular architecture. Computed tomography 3D reconstructions of a barium perfused *en block* preparation of the patella; the patellar ligaments; and the tibial tuberosity (**A** cranial view; **B** caudolateral view; **C** caudal view) and the infrapatellar fat pad (**D** cranial view) of case no. 3; perfused vessels are seen in white. Each ligament received a bipolar vascular supply with a high vessel density at the poles, from which numerous distinct longitudinal vessels coursed distally or proximally, until anastomosing in the middle third within each ligament. One distinct longitudinal vessel was seen entering each of the lateral and medial lobes of the infrapatellar fat pad at the distocranial aspect, branching into extensive vascular plexus within each lobe. The medial and lateral plexus was connected with several horizontal branches after the two lobes merged. **E** Photomicrograph of the IFP illustrating intravascular barium (dark pigment) at 2.5 × magnification. H&E staining

The technical quality of the barium perfusion was deemed adequate for analysis in all perfused limbs as there were no signs of overfilling and/or barium leakage. Histology demonstrated presence of intravascular barium in all three ligaments and in the IFP (Fig. 1).

In all specimens, the main vascular architecture represented by barium-filled vessels followed similar trajectories and branching patterns in both the PLs and in the IFP as illustrated in Fig. 1. The PLs received a bipolar blood supply; vessels supplying the PLs from the proximal aspect were identified as branches of the proximal genicular and descending genicular arteries, whereas vessels supplying the PLs from the distal aspect were identified as branches of the distal genicular artery [15]. There was a high vascular density at the poles of each ligament, from which numerous distinct longitudinal vessels coursed distally or proximally, until anastomosing in the middle third within each ligament; these vascular anastomoses were more numerous in the intermediate versus the medial and lateral PLs. Longitudinal vessel number and the proportion of centrally located vessels per ligament are reported in Table 1. An extensive latticework of smaller vessels was seen throughout all ligaments. There were also several vascular connections between the PLs and the IFP, with larger vessels predominantly found in the distal and proximal thirds of the ligaments at the caudomedial, caudolateral and the craniolateral aspects of the lateral, intermediate and medial PLs, respectively.

**Table 1:** Number of distinct longitudinal vessels (mean ± SD) identified on 2D transverse CT images of barium perfused specimens reported for proximal, middle and distal locations for all three patellar ligaments; and proportion of centrally located vessels per ligament

	**Proximal**	**Middle**	**Distal**	**Proportion central vessels**
	**Vessel no**	**Vessel no**	**Vessel no**	
**Lateral PL**	13 ± 4	11 ± 4	15 ± 9	55%
**Intermediate PL**	13 ± 4	11 ± 5	10 ± 4	44%
**Medial PL**	15 ± 4	12 ± 4	13 ± 7	48%

Vessels supplying the IFP were identified as branches of the descending genicular artery [15]. The IFP was highly vascularized (Fig. 1); one distinct longitudinal vessel was seen entering each of the lateral and medial IFP lobe at the distocranial aspect, immediately branching into an extensive vascular plexus supplying each lobe until anastomosing by several horizontal branches where the two lobes merged. In total, 63 ± 11 larger vessels were observed proximally; 74 ± 9 in the mid portion; and 54 ± 20 in the distal portion of the pad.

### Histology

**Fig. 2:**
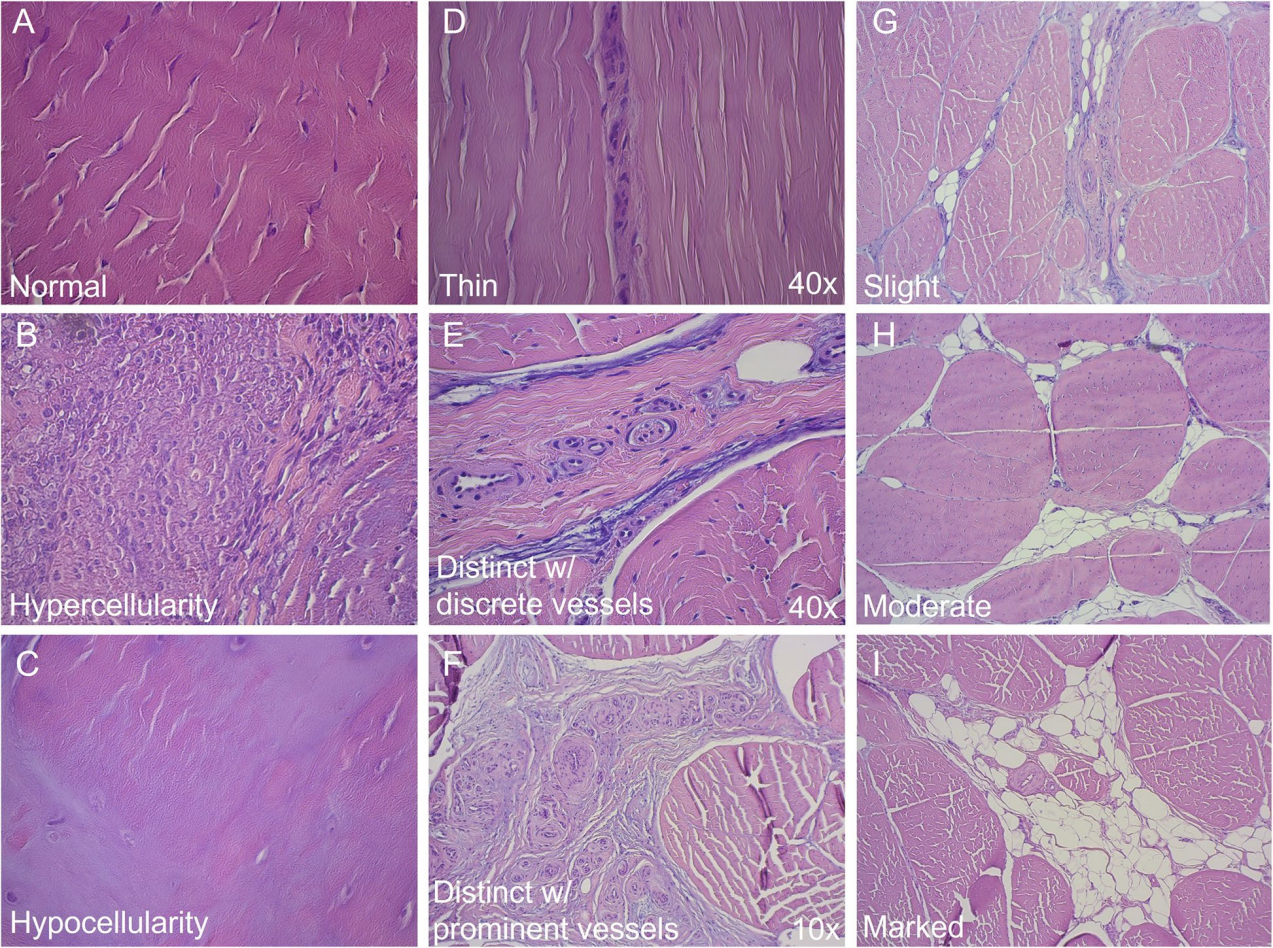
Histological characteristics of patellar ligaments. Photomicrographs illustrating characterization of patellar ligament cellularity (**A**—**C**; all obtained at 40 × magnification); endotenon thickness and vascularity (**D** – **F**); and endotenon fatty infiltration (**G** – **I**; all obtained at 10 × magnification). **A** Normal fascicle cellularity; scattered tenocytes are seen within the fascicles. **B** Hypercellularity; areas of tenocyte clustering. **C** Hypocellularity; areas almost devoid of cells. Areas of hypocellularity coincided with areas of metaplastic tenocytes / chondroid metaplasia. **D** Thin endotenon (40 × magnification). **E** Distinct endotenon with discrete vessels (40 × magnification). **F** Distinct endotenon with prominent vessels (10 × magnification). **G** Slight endotenon fatty infiltration. **H** Moderate fatty infiltration. **I** Marked fatty infiltration

**Fig. 3:**
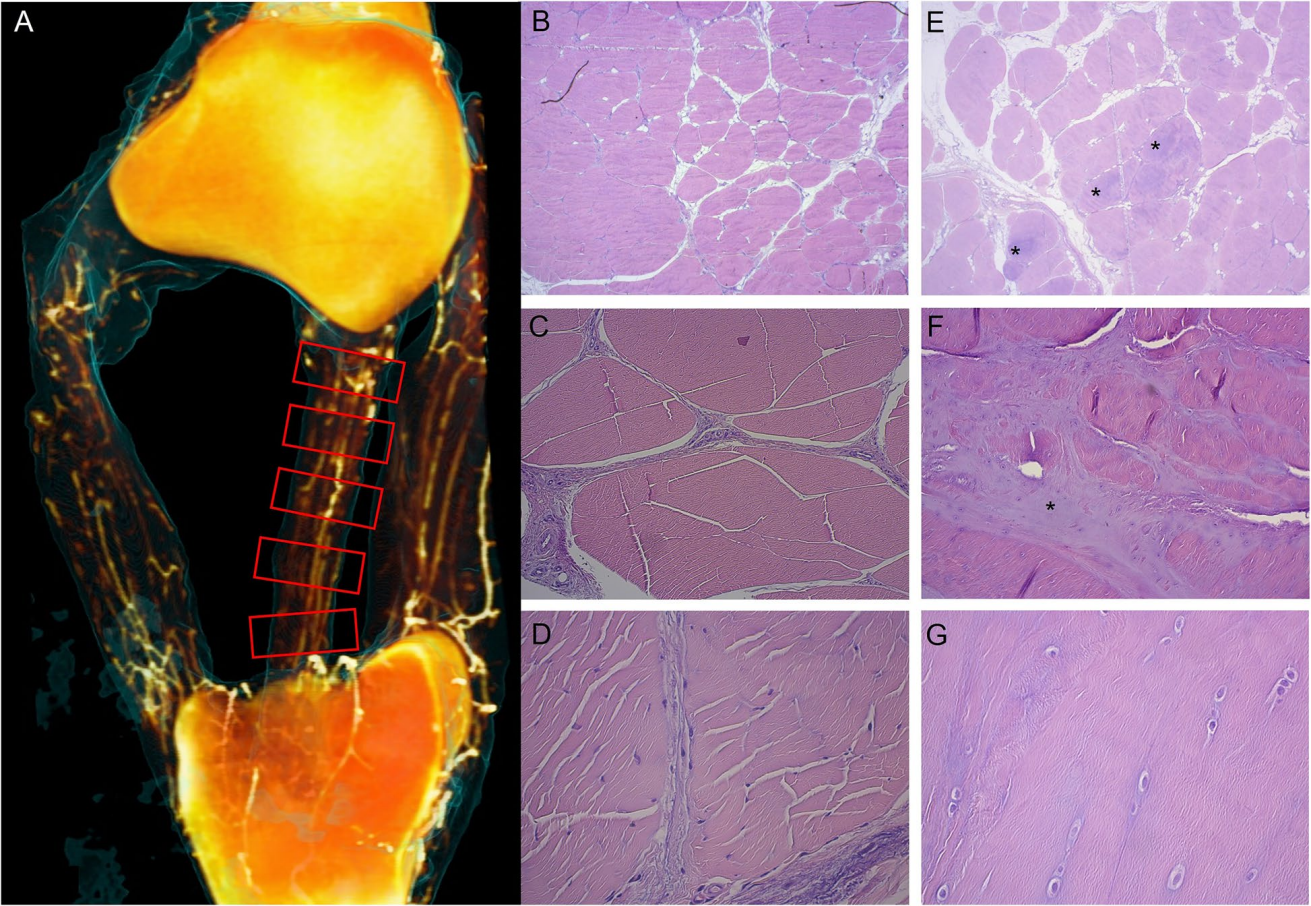
Sampling sites and normal ligament structure versus metaplastic tenocytes / chondroid metaplasia. Caudal view 3D reconstruction of a barium perfused *en block* preparation of the patella; the patellar ligaments; and the tibial tuberosity of case no. 2; perfused vessels are seen in white; the ligament outline is seen in blue. Red boxes illustrate the location for the tissue blocks harvested from each ligament, here illustrated for the intermediate patellar ligament. **B-D** Photomicrographs illustrating normal ligament structure at 2.5x, 10 × and 40 × magnification, respectively. Polygonal fascicles consisting of collagen bundles with scattered tenocytes are seen separated by connective tissue septa (endotenon) carrying blood vessels, lymph vessels and nerves. Tenocyte nuclei are spindle shaped (type 1). **E**–**G** Photomicrographs illustrating metaplastic tenocytes / chondroid metaplasia at 2.5x, 10 × and 40 × magnification, respectively. The normal extracellular matrix has been replaced by chondroid matrix (asterisks), and tenocyte nuclei are rounded, located in semi-lacunae

Due to sectioning artefacts, 7 of the total 105 H&E ligament Sects. (1 section from the medial PL; and 3 each from the intermediate and lateral PL) were not of diagnostic quality and were subsequently omitted from all analyses. All adult ligaments consisted of polygonal fascicles separated by endotenon septa of varying thickness carrying a variable amount of blood vessels, lymph vessels, nerves and fat (Fig. 2). In all investigated sections from the adult horses, the majority of fascicles had normal cellularity populated by predominantly type 1 tenocytes. Smaller areas with metaplastic tenocytes / chondroid metaplasia, defined as areas of hypocellularity, were identified in the medial PL in all adult horses and throughout the ligament length (Fig. 3). Areas of metaplastic tenocytes were also identified in the intermediate PL in 2/7 and in the lateral PL in 4/7 horses; in both ligaments, this finding was seen in all sections except at the ligament origin. Toluidine blue staining of these sections revealed a darker blue staining indicating an increased glycosaminoglycan content of the extracellular matrix. Hypercellularity with clustering of tenocytes was seen in 2/7 horses in the lateral PL, in 1/7 in the intermediate PL, and in 1/7 horse in the medial PL.

The intermediate and the medial PL of the foal displayed a polygonal fascicular architecture with a slightly higher cellularity than seen in the adult ligaments however mainly populated by type 1 tenocytes. In the lateral PL, a polygonal fascicular architecture was seen at the cranial and peripheral aspect of the ligament, whereas continuous intra- and interfascicular endotenon septa had yet to form at the caudal aspect where clusters of vessels were seen embedded within fascicular tissue but not within endotenon septa. Also, all investigated lateral PL sections from this young animal displayed hypercellularity, predominantly populated by type 2 tenocytes. Metaplastic tenocytes or adipose tissue were not seen in any of the PLs in this animal.

Interfascicular endotenon characteristics were significantly associated with age for the intermediate and medial PL (*P* < 0.0001 for both, respectively), but not for the lateral PL (*P* = 0.09). In the intermediate PL, a thin endotenon was observed in younger animals (≤ 6 years of age); a distinct endotenon with discrete vessels was observed across all ages, whereas a distinct endotenon with prominent vasculature was seen in the older animals (≥ 6 years of age). In the medial PL, the interfascicular endotenon was thin in animals younger than 6 years whereas a distinct endotenon with discrete vessels was consistently seen in animals 13 years and older; a distinct endotenon with prominent vasculature was not observed in this ligament. In the lateral PL, the most common observation was that of a distinct endotenon with discrete vasculature, and no association with age was found. When assessed objectively, the interfascicular endotenon of the medial PL (mean 39 µm, 95% confidence interval 37 – 40 µm) was significantly thicker than that of the intermediate (mean 27 µm, 95% confidence interval 26 – 28 µm) and the lateral PL (mean 21 µm, 95% confidence interval 19 – 23 µm), *P* < 0.001; and the endotenon of the intermediate PL was significantly thicker than that of the lateral PL (*P* < 0.001).

Fatty infiltration was significantly associated with age for the intermediate and medial PL (*P* = 0.0017, and *P* = 0.02, respectively), but not for the lateral PL (*P* = 0.1). In the intermediate PL, none or slight fatty infiltration was seen in younger animals, whereas slight or moderate fatty infiltration was seen in older animals. In the medial PL, slight fatty infiltration was seen across all age groups, whereas moderate fatty infiltration was predominantly seen in the older animals. The lateral PL had the least amount of fatty infiltration, with none or slight infiltration seen across all age groups.

All IFP sections were considered to be of diagnostic quality. In all adult horses, the IFP consisted of white unilocular adipose tissue, organized in lobules separated by thin connective tissue septa measuring 4 – 60 µm in width (mean 20 µm; median 15 µm); septal thickness increased towards the periphery and towards the distocentral aspect; in these areas, septal thickness had a mean of 113 µm and a median of 90 µm. Mean lobular diameter was 590 µm (median 544 µm). Inflammatory cell infiltrates were not identified in any section. The IFP of the foal consisted of a mix of white unilocular adipose tissue and brown multilocular adipose tissue organized in lobules separated by thin connective tissue septa. Synovial hyperplasia, lymphocytic infiltration, fibrosis or mucoid change was not observed in the synovial membrane in any of the investigated sections in which the synovial membrane was preserved.

## Discussion

Findings of the current study demonstrated that all three PLs were extensively vascularized similar to what previously has been reported for the superficial digital flexor tendon [11].

The arterial supply to the equine PLs derives from proximal and distal sources [15]. As the three PLs originates from different aspects of the patella, a distinct retropatellar vascular arch as described in humans [16] was not seen, instead, the origin of each ligament appeared highly vascularized. However, as the PLs converge at their insertion, the high vascular density at the distal ligament poles correspond to the supratubercular vascular arch in humans [16]. Horizontal vascular anastomoses were more numerous in the middle third of the intermediate versus the middle third of the lateral and medial PLs, most likely reflecting different physiological demands. In the human patellar tendon, the posterior region, which is the site most commonly affected by tendinopathy, is less vascularized than the anterior aspect as it receives its blood supply from the IFP rather than from the genicular vascular anastomoses supplying the anterior region [17]. A less vascularized zone was not identified in the equine intermediate PL, which biomechanically is the ligament most likely at risk for developing an overuse tendinopathy similar to the clinical condition in human athletes. Several vascular connections were observed between the IFP and all three ligaments, and contrasting humans, such connections were predominantly seen in the distal and proximal third of the ligaments and were thus located in areas with ample concurrent vasculature. As in humans, we observed direct neurovascular links between the IFP and the PLs suggestive of crosstalk between these structures. Release of proinflammatory cytokines from the IFP has been suggested to contribute to patellar tendinopathy in humans [14]. The role of this structure in stifle pathology in horses has yet to be determined.

Although there was considerable variation in the amount of endotenon vascularity, there were no histological signs of neovascularization in the horses studied as vessels were confined to the interfascicular and intrafascicular endotenon. Thus, identifying discrete PL vessels by ultrasonography should be interpreted with caution as these might represent normal ligamentous endotenon vascularity. The vessel clusters seen within the fascicular tissue in the caudal aspect of the lateral PL in the foal were interpreted as a representation of immaturity rather than pathology. Interestingly, both the medial and the intermediate PLs of the foal had a mature architecture of polygonal fascicles, illustrating a difference in maturity between the different ligaments.

There was a large range in interfascicular endotenon thickness, which increased with age for the intermediate and the medial PL. Age related interfascicular endotenon changes have previously been reported for the equine superficial digital flexor tendon [18]; the canine cranial cruciate ligament [19]; and for the canine patellar tendon [20]. Although the stiffness of the canine patellar tendon under physiologic loading did not change with age, the total collagen concentration decreased significantly with age [20]. Total collagen concentration was not measured in the current study. However, a thicker endotenon as observed in the older animals indicates a lower collagen proportion given a consistent ligament thickness and could potentially be responsible for some of the heterogenicity and striations commonly observed ultrasonographically in equine PLs. The finding of a thicker endotenon with increasing age, contradicts previously reported findings of the equine superficial digital flexor tendon where the mean thickness of the interfascicular endotenon in the distal region decreased with age [18], rendering the tendon more vulnerable to overuse injury as the interfascicular tissue plays a major role in allowing fascicle sliding during load [21]. This discrepancy points towards structure-specific age-related changes, most likely due to different physiological properties of the structures investigated. Although the biomechanical characteristics of the equine PLs have yet to be fully investigated, the low overuse injury rate compared to for example the superficial digital flexor tendon indicates that these structures usually are working within their biomechanical capacity; alternatively, that the thicker endotenon may offer some protective effect towards strain-related injury with age. Indeed, a thick endotenon with discrete vessels was consistently seen in the medial PL of the older animals in the current study, and is therefore interpreted as a normal, age-related finding. However, a prominent vasculature was seen in the thick endotenon of the intermediate PL in older animals and could be interpreted as a sign of (subclinical) PL repair or degeneration. However, the significance of this finding is currently unknown.

The development of metaplastic tenocytes / chondroid metaplasia is proposed to be the first abnormal histological finding in human patellar tendinopathy, preceding extracellular matrix changes and neovascularization [22]. Chondroid metaplasia is however also a physiological adaptation in tendons subjected to compressive loads, typically in tendons wrapping around bony pulleys [23]. Chondroid metaplasia has been reported as biologic variation in equine PLs at or near points of insertion [24] and is regarded as age-related changes of the intra-articular soft tissue stifle structures [25]. Areas of chondroid metaplasia were found in all investigated medial PLs in the current study and was therefore interpreted as normal findings within this ligament, possibly due to its specialized function in the passive stay apparatus. We observed chondroid metaplasia not only at the points of insertion but also in the mid body of this ligament, and in animals as young as 3 years of age suggesting that rather than age-related, these changes may be physiological adaptations. Moreover, areas of chondroid metaplasia and increased GAG content could be responsible for some of the heterogenic echogenicity commonly observed upon ultrasonographic examination of the equine PLs, which especially when detected in the medial PL, should be interpreted with care. Another finding which could result in heterogenic ultrasonographic appearance is fatty infiltration, which in the current study increased with age for the medial and intermediate PLs. Indeed, fatty infiltration resulted in abnormal magnetic resonance imaging signal intensity of the PLs of equine cadaver stifles, although the magnitude of fatty infiltration was not described [25].

Coinciding with descriptions from human medical literature [13], the equine IFP was highly vascularized and consisted of lobules of white adipose tissue characterized by a significant amount of fibrous stroma, which is typical for areas subjected to considerable mechanical stress. The fibrous septa were thicker in central areas of the fat pad, and thinner towards the periphery. There were no signs of inflammatory infiltrates of the IFP of the horses in the current study. However, the rich vascular supply infers a biologically active tissue, which potentially could contribute to joint pathology in horses similar to what has been described in humans. Thus, the role of the equine IFP in joint disease should be investigated further.

Result from the current study demonstrate that equine PLs with no clinical or ultrasonographic signs of injuries display considerable histological changes. The heterogenous appearance and indistinct margins of the lateral PL observed in two of the included cases was interpreted as normal anatomical variation in line with previous reports [3]. A mottled or striated ligamentous insertion identified in the transverse plane, as well as a fan-like ligamentous insertion in the longitudinal plane, was also interpreted as normal variation as previously reported in normal horses [1, 3]. Ultrasonographic changes seen in included horses were symmetric and therefore not considered to represent injury. The presence of bilateral injury could not be completely excluded but was considered unlikely due to the lack of concurrent clinical findings such as lameness and joint effusion. Our data suggests that PL ultrasonography is not sensitive enough to detect the histological variation in vascularization and tissue characteristics identified and described above. It is however likely that ultrasonographic changes interpreted as pathological PL lesions would translate into other histopathological findings and future histopathological validation of PL injuries could potentially improve our understanding of the relevance of ultrasonographic findings of the equine PLs and help discriminate between normal age-related changes and clinical disease.

A limitation of the study was the use of grayscale B-mode ultrasonography only, as this modality is less sensitive than color doppler or power doppler techniques in detecting blood vessels. However, previous studies have reported that power Doppler signals indicating neovascularization are not detected in structures such as suspensory ligament branches and the superficial digital flexor tendon when there are no abnormal findings detected by B-mode ultrasonography [10, 26]. The fact that power Doppler signals were also seen in suspensory ligament branches of sound limbs albeit with abnormal B-mode ultrasonographical findings [26], complicates the interpretation of the clinical relevance of such findings and highlights the need for histopathological confirmation of disease. Another limitation of the study was the cross-sectional nature of the study and the limited number of research subjects, which was a convenience sample of horses euthanized at the institution during the study period. A larger cohort of horses with more ages represented would have been preferable.

## Conclusion

The equine PLs and IFP are highly vascularized structures; the PL vascular architecture is similar to that of the superficial digital flexor tendon. This, together with the large variation in endotenon thickness and vascularity, should be taken into consideration when assessing potential patellar ligament histopathology. Endotenon thickness and fatty infiltration is increasing with age for the medial and intermediate PLs, but not for the lateral PL. Metaplastic tenocytes / chondroid metaplasia should be considered a normal finding throughout the medial PL, and in all ages.

## Methods

A convenience sample of tissues from horses euthanized at the institution for reasons other than stifle disease was used. The main PL and IFP vascular pattern was described based on computed tomography (CT) scanning of barium perfused specimens, whereas the histological architecture was described from tissue processed with H&E and toluidine blue staining.

### Horses

Upon obtaining informed consent from animal owners, an opportunistic cohort of one 10-day old foal and seven adult horses (age 3 – 18 years) euthanased at the institution for reasons unrelated to stifle pathology were recruited to the study; case details and reasons for euthanasia are detailed in Supplementary table 1. The study was approved by the ethical review board at the institution and was in accordance with national legislation regarding use of animals in research (FOR-2015–06-18–761).

Prior to euthanasia, adult horses were subjected to a standardized lameness examination including walking and trotting in a straight line on a hard surface and were included in the study when no baseline hind limb lameness was detected (defined as AAEP grade 0). The stifle area was palpated for soft tissue swelling such as joint effusion and periligamentous thickening. A standardized B-mode ultrasonographic examination (Esaote MyLab Gold ultrasonography machine, using a multifrequency linear probe set at 10–15 MHz) of the stifle was performed in a weightbearing position. In brief; following a routine skin preparation including hair removal (size #40 clipper blades) and application of alcohol and coupling gel, the medial, cranial and lateral aspect of each stifle was examined in longitudinal and transverse planes. Structures evaluated at the medial aspect included the tibial plateau; medial meniscus and medial collateral ligament. Presence or absence of medial femorotibial joint effusion; osteophyte formation; and ligamentous or meniscal injuries were recorded. At the lateral aspect, the lateral collateral ligament; lateral meniscus and lateral femorotibial and femoropatellar synovial pouches were assessed for the same abnormalities. The cranial aspect of the stifle was then evaluated focusing on the intermediate, medial and lateral PLs as well as the medial and lateral femoral trochlea and the trochlear groove. The three patellar ligaments were scanned in transverse and longitudinal planes from their origin to the tibial insertions. Ligament shape, size, echogenicity and fiber pattern was evaluated and considered normal when findings were in line with previously reported literature [1–6]. Abnormal findings such as deviations in size, shape and/or echogenicity pattern were noted and considered normal if findings were bilaterally symmetric.

Included animals were administered i.v. heparin (500 IU/kg) as an adjunct to the routine euthanasia protocol which consisted of premedication with detomidine / butorphanol, anesthesia induction with ketamine/ midazolam, followed by an overdose of pentobarbital. Immediately after euthanasia, one randomized hind limb was subjected to a barium perfusion procedure and subsequent CT scanning, while the contralateral hind limb was used for histology only. Histology was also obtained from two barium perfused limbs after CT scanning, to validate that the barium identified on the CT scans was located intravascularly, thus representing blood vessels.

### Barium perfusion and CT scanning

Limbs were harvested at the coxofemoral joint and suspended from the distal limb for 3 h prior to being subjected to barium perfusion using modifications of a previously published protocol [27]. In brief, this protocol includes stepwise perfusion of the limb via the femoral artery with 1) isotonic saline until the effluent runs clear; 2) 20% v/v micronized barium suspended in saline; and finally, 3) 20% v/v micronized barium suspended in 10% neutral buffered formalin (NBF). Perfused limbs were refrigerated for 48 h to allow the barium to set in the arterial vasculature prior to *en block* harvesting of the patella, the PLs and the IFP for further tissue fixation (10% NBF for a minimum of 48 h followed by ethanol) and subsequent CT scanning. Tissue harvesting was achieved by sharp dissection of periarticular soft tissues from the patella and PLs, taking care to separate the caudodistal portion of the medial PL from the common aponeurosis of the gracilis and sartorius muscles, and to separate the lateral aspect of the lateral PL from the aponeuroses of the biceps femoris and the fascia lata muscles [28]. The tibial tuberosity was cut from parent bone using a bone saw: a transverse cut was placed immediately cranial to the menisci, and a horizontal cut was placed distal to the tibial tuberosity, taking care to leave all PL insertions intact. In the foal and one adult horse, the IFP was left intact with the *en block* specimens for tissue fixation and scanning, whereas in 6/7 adult horses, the IFP was removed by sharp/blunt dissection from the intermediate PL for separate fixation and scanning.

CT scanning was performed using a 4-slice GE CT scanner (GE Medical Systems Bright Speed S); slice thickness was 0.625 mm. The digital CT images were subsequently processed using ImageJ and Osirix Dicom 3D imaging software. The technical quality of the barium perfusion was assessed and deemed of adequate quality for analysis when perfused vessels were clearly visible within each of the PLs; the patella; and within the tibial tuberosity. Potential perfusion artefacts (overfilling and/or barium leakage) was recorded. Quantitative and qualitative assessments of perfused vessels commenced. For all PLs, the main intraligamentous vessel morphology including trajectory and branching pattern was described from 3D reconstructions; for the specimens in which the IFP was left in place for the scan, the vascular association between the IFP and intermediate PL was described. Within each ligament, the number of distinct, barium-filled longitudinal vessels were counted on 2D transverse images at 3 different locations (proximally, defined as immediately distal to the patellar origin; at the mid-portion, halfway between the patella and the tibia; and distally, immediately proximal to the tibial insertion), and the vessel cross-sectional location (central vs. peripheral) was recorded and reported as the proportion of centrally located vessels. In the IFP, the number of distinct, barium-filled vessels were counted on 2D transverse images at 3 different locations; 1/3 from the proximal pole; at the mid portion; and 1/3 from the distal pole.

### Histology

*En block* harvesting of the patella, PLs, IFP and tibial tuberosity of the contralateral limb was performed as described above and placed in 10% NBF for 48 h. Five 5 mm tissue blocks were sharply cut perpendicular to the longitudinal fiber orientation of each ligament as illustrated in Fig. 3, from the origin at the patella/medial parapatellar fibrocartilage, to the insertion at the tibial tuberosity, taking care not to include bone and/or cartilage at the ligament origin/ insertion. Five 5 mm tissue blocks were sharply cut from the IFP, perpendicular to its longitudinal axis. Tissue blocks were embedded in paraffin and 4—6 µm sections were routinely processed with H&E staining. Tissue blocks in which metaplastic tenocytes were identified in H&E sections were also processed with toluidine blue staining. Sections were evaluated at 2.5x, 10 × and 40 × magnification by light microscopy and photographed using a Zeiss™ digital photomicroscope (AxioCam ERc5s) and then copied and stored onto a computer using designated software (Zen blue, Carl Zeiss).

All sections were assessed for diagnostic quality, and sections in which ligament structure and cellular morphology could not be assessed due to processing artefacts were omitted from the analyses. Normal ligament structure was defined as polygonal fascicles consisting of collagen bundles with scattered tenocytes, separated by endotenon recognized as interstitial connective tissue septa carrying blood vessels, lymph vessels and nerves, as illustrated in Fig. 3. Tenocytes were classified as normal when nuclei were spindle shaped (type 1) or when nuclei were plump and cigar shaped (type 2). Tenocytes with rounded nuclei situated in semi-lacunae were defined as abnormal and referred to as metaplastic tenocytes. Tissue vascularity was defined as normal when blood vessels were confined to the endotenon and abnormal when vessels were seen within fascicles.

The interfascicular endotenon was subjectively classified as thin when it was 1–2 cell layers thick, and as distinct when it consisted of multiple cell layers; the distinct interfascicular endotenon were further characterized as having a predominantly discrete or prominent vascularity as illustrated in Fig. 2. Interfascicular endotenon thickness was objectively measured on ruler-calibrated images captured at 2.5 × magnification using imaging software (ImageJ); cross-junctional areas of the endotenon were excluded from the analysis as they formed irregular structures.

Fascicle cellularity was defined as normal when scattered tenocytes were seen within the fascicles, whereas hypercellularity was defined as areas of tenocyte clustering (Fig. 2). Contrary, hypocellularity was defined as areas almost devoid of cells, which coincided with areas of metaplastic tenocytes (Fig. 2).

Ligamentous fatty infiltration was defined as normal when adipocytes were confined to the endotenon, and as abnormal when intra-fascicular adipocytes were seen. When confined to the endotenon, the amount of fatty infiltration was subjectively graded as none; slight; moderate or marked as illustrated in Fig. 2.

In the toluidine-blue sections, the extracellular matrix glycosaminoglycan content was subjectively assessed as increased in areas of increased basophilia.

Normal histological architecture of the IFP was defined as lobes of adipose tissue separated by connective tissue septa, with a synovial membrane lining along its caudal aspect and with no presence of inflammatory cell infiltration. Interlobular connective tissue septa were subjectively assessed as thin or distinct; septal thickness as well as lobular diameter were objectively measured in images acquired at 2.5 × magnification using imaging software (Image J).

### Statistics

Descriptive statistics were applied to all measured parameters. Due to non-normal distribution, the interfascicular endotenon thickness measurements were compared between ligaments using the Wilcoxon method. For each ligament, the association between age and endotenon characteristics and fatty infiltration was tested using univariable logistic regression analyses; model fit was assessed with the effects likelihood ratio test. Significance was defined as *P* < 0.05 for all analyses.

## Supplementary Information

**Additional file 1:** **Supplementary Table 1.** Case details and reasons for euthanasia for included animals.

## Data Availability

Data are available from the corresponding author upon reasonable request.
