# Supplementary material for: The equine patellar ligaments and the infrapatellar fat pad – a microanatomical study
Source: BMC Vet Res. 2023 Jan 23;19:20. doi: 10.1186/s12917-023-03579-3 (PMC9869593; doi:10.1186/s12917-023-03579-3)
Supplement: Supplementary file 1 — Additional file 1: Supplementary Table 1. Case details and reasons for euthanasia for included animals. [file 12917_2023_3579_MOESM1_ESM.docx]

| **Case no** | **Age (years)** | **Breed** | **Sex** | **Reason for euthanasia** |
| --- | --- | --- | --- | --- |
| 1 | 10 days | NSCT | m | Unwanted covering |
| 2 | 3 | STB | m | Bilateral fore limb lameness, DIPJ OA |
| 3 | 4 | STB | f | SDTF tendonitis LF |
| 4 | 6 | STB | f | Chronic forelimb lameness |
| 5 | 6 | STB | m | Chronic diasthema |
| 6 | 13 | STB | g | Chronic forelimb lameness |
| 7 | 15 | STB | f | Retired brood mare |
| 8 | 18 | Welsh Pony | f | Cardiomyopathy |

**Supplementary Table 1.** Case details and reasons for euthanasia for included animals. NSCT = Norwegian-Swedish Coldblooded Trotter; STB = Standardbred. m = male. f = female. g = gelding. DIPJ = distal interphalangeal joint. OA = osteoarthrosis. SDFT = superficial digital flexor tendon. LF= left front limb.
